# Joint modeling of forced vital capacity measures with time to onset of polycythemia among chronic obstructive pulmonary outpatients follows‐up: A case of University of Gondar Referral Hospital

**DOI:** 10.1002/hsr2.1587

**Published:** 2023-09-28

**Authors:** Yoseph Kassa, Dessie Melese, Anteneh Asmare, Gashu Workneh

**Affiliations:** ^1^ Department of Statistics, College of Natural and Computational Science Oda Bultum University Chiro Ethiopia; ^2^ Department of Statistics, College of Natural and Computational Science University of Gondar Gondar Ethiopia

**Keywords:** chronic obstructive pulmonary patients, Cox‐proportional hazard model, forced vital capacity, joint model, linear mixed model, time to onset of polycythemia

## Abstract

**Background and Aims:**

Chronic obstructive pulmonary disease (COPD) causes airflow obstruction and respiratory problems. Thus, the main objective of this study was to determine the risk factors for the progression of COPD using longitudinally measured forced vital capacity with time to onset of polycythemia outpatients follow‐up.

**Methods:**

A retrospective study design was used to gather the related data on longitudinal change of forced vital capacity and time to onset of polycythemia from the medical charts. The joint model consists of a longitudinal submodel for the change of forced vital capacity and a survival submodel for the time to onset of polycythemia of chronic obstructive pulmonary patients.

**Results:**

From the total of 266 patient's estimated value of forced vital capacity of chronic obstructive pulmonary patients was 74.45 years with a standard deviation of 8.59. The estimated value of the association parameter was −0.006, which indicates that the lower value for a forced vital capacity measure was associated with the higher risk of polycythemia and vice versa “Based on the joint model analysis found that the predictor smoking, comorbidities, marital status, weight, and HIV” jointly affected the two responses, which are change of forced vital capacity and time to onset of polycythemia among chronic obstructive pulmonary patients.

**Conclusion:**

The overall performance of separate and joint models, joint modeling of longitudinal measures with the time‐to‐event outcome was the best model due to smaller standard errors and statistical significance of both the association parameters.

## 1 BACKGROUND

Chronic obstructive pulmonary disease (COPD) is a condition that can be prevented and treated and is characterized by difficulty breathing that isn't entirely curable. COPD is the third leading motive of death globally, affecting 3.23 million deaths in 2019.^1^

Globally, the maximum typically encountered chance aspect for COPD is tobacco smoking. Other types of tobacco and marijuana also are chance elements for COPD. Outside, occupational, and indoor air pollutants—the latteras a result of the burning of biomass fuels are the major principal COPD risk factors.^2^

Noncommunicable diseases and chronic respiratory disease, the key cause of death and morbidity globally. The most recent suggestion is accessible from the Global Burden of Disease Study 2017 reports. Around 3.2 million deaths due to (COPD) and 495,000 deaths due to asthma.^3^

The incidence of COPD is increase due to urbanization, industrial pollution, tanneries, and biomass fuel burning inside homes, particularly in Asian and African countries. The prevalence COPD in Africa is 13.4%, ranging from 9.4% to 22.1%. But in Asia, its prevalence was reported at 13.5% with a range of 3%−22.2%. Additionally, COPD is a common cause for hospital admission in many countries, playing a crucial role in imposing healthcare costs.^4^

Some study showed that the occurrence of COPD in sub‐Saharan Africa has been decreased. But, COPD has turned out to be a growing health problem in sub‐Saharan Africa since of tobacco smoking and the publicity of biomass fuels. In the most worldwide location of sub‐Saharan Africa, 90% of agricultural household determined by on biomass gasoline for cooking and heating, determine younger kids (severe decreased breathing infections) and ladies (COPD). It is the reason of large death and illness in the vicinity.^5^

Some of understanding the relationship between infected with HIV and COPD in sub‐Saharan Africa. We evaluated the occurrence and studied the risk factors of COPD consistent with HIV status in a reference center for HIV and tuberculosis control in Cameroon.^6^

Around 63.3% of the contributors with COPD provided with cough as the primary respiration sign. Nearly 55.5% of men patients with COPD and 45.6% of women patients with COPD had a cough. Breathing signs of cough, phlegm, wheezing, and rapidity of breath had been significantly more usual in patients with COPD than people who no longer have COPD.^7^

The report discussed above shows that up‐to‐date assessment of the evidence concerning COPD in Ethiopia is very low in the documentation at in country level. COPD is increasing from time to time and that affects developing countries like Ethiopia. The majority of population deaths, according to the experts, are brought on by chronic obstructive lung disorders. This implies that further studies are needed to identify and evaluate factors or prevalent methods for the progression of forced vital capacity with time to onset of polycythemia among chronic obstructive patients under the follow‐up. The prevalence of COPD increases with age and people are not normally recognized till they may be 50 years of age or older. It is far extra usual in male than female and men are much more likely to die from it. Mortality prices are larger in Scotland and the North of Britain than in the South, reflecting the fact that the prevalence and its consequence are nearly two times as high within the most deprived 20% of the population.^8^

This finding observed that there is an increasing progression of COPD in our country. The researcher was find out a way of handling to minimize and guide the prevalence of the diseases by using repeated measurement of forced vital capacity with the onset of polycythemia due to the follow‐up among COPD. An investigation was conducted based on a cross‐sectional study design. This study design does not necessarily show the prevalence of disease over time. They used logistic regression to determine the risk influences without allowing for the relationships within the many outcomes and subject‐specific random effects. In this investigation, such kind of problem was solved by considering the correlated and missing data in the longitudinal submodel.

The linear mixed model for longitudinal forced vital capacity and the Cox proportional hazard (PH) model for to time to onset of polycythemia data do not study dependences or relationships between these two different data types (longitudinal and time‐to‐event data) independently. Therefore, the researcher had an increasing interest in joint modeling of longitudinal forced vital capacity measure with time to onset of polycythemia in chronic obstructive pulmonary outpatients. Then it minimizes unfairness in parameter approximation and increase the adequacy of statistical inference. Therefore, the main objective of this investigation was to determine the determinant risk factors for the progression of COPD using longitudinally measured forced vital capacity with time to onset of polycythemia outpatients follow‐up.

## 2 METHODS

### 2.1 Study area

The investigation has conducted at University of Gondar Referral Hospital. University of Gondar Referral Hospital is one of the oldest institutions of in Ethiopia. It has been producing a number of health workers of science more than half of a century ago. The university is situated at the center of Gondar city found in Amahara Region, North West of Ethiopia. The hospital provides different inpatient and outpatient services to the population in the surrounding area of Gondar town and the nearly by Woredas and Zones. Gondar town is the capital city of Central Gondar zone and located 727 km away from Addis Ababa in the northwest of Ethiopia, Amahara Region.

### 2.2 Data source and study design

For this research, a secondary source of data was considered. The study designs for this study were an observational retrospective study. The patient's chart using a checklist designed by the researcher by considering available variables, which includes clinical and sociodemographic data on all the COPD outpatients during follow‐up, was used to extract both longitudinal and survival data. The study period was from February 1, 2019 to February 1, 2022, a total of 266 chronic obstructive pulmonary outpatients were obtained in the University of Gondar Referral Hospital. The data for responses and covariates were collected with the support of the healthcare service. In this study, chronic obstructive pulmonary outpatients represent the number of patients who follows the clinical treatment up to the discharge date, and or who either leave the hospital by any means or transfer from the hospital to the other hospital or die before completing the treatment.

### 2.3 Inclusion and exclusion criteria

#### 2.3.1 Inclusion criteria

The study population was all chronic obstructive pulmonary outpatients who have two or more visits and patients measured whose, forced vital capacity in the follow‐up were included in the study period.

#### 2.3.2 Exclusion criteria

Chronic obstructive pulmonary outpatients who have only single information and patients not measure the forced vital capacity were excluded from the study.

### 2.4 Study variables

#### 2.4.1 Outcome variables

Two outcome variables were considered, these are: The longitudinal measures outcome: progression of forced vital capacity measured with a spirometer approximately every 3 months. It is a continuous variable. The survival outcome: time to onset of polycythemia for chronic obstructive pulmonary outpatients (0 = *censored* & 1 = *event*).

#### 2.4.2 Independent variable

The explanatory variable for this investigation were sociodemographic or clinical characteristics that are expected to be related to repeated measurements of forced vital capacity with time to onset of polycythemia in chronic obstructive pulmonary outpatients during treatment shown in Table 1 below.

**Table 1:** Independent variable.

No	Variables		Description	Codes
1	Visit time		Observation times (in a month)	
2	Sex		Sex of patient	0 = Female, 1 = male
3	Age		Age of patient (in years)	
4	Residence		Residence of patient	0 = Rural, 1 = urban
5	Weight		Weight of patient	
6	FVC		Forced vital capacity (spirometer)	
7	Marital		Marital status of patients	0 = Single, 1 = married, 2 = widowed 3 = divorce
8	Smoking		Patients Smoking status	0 = Nonsmoker 1 = smoker
9	Education		Education level of patients	0 = No educated, 1 = primary educated, 2 = secondary educated, 3 = diploma and above
10	Comorbidities		Presence of related diseases	0 = No, yes = 1
11	HIV		HIV status of patients	No = 0, yes = 1
12	Occupation		Occupation of patients	0 = Farmer, 1 = merchant, 2 = government worker, 3 = others

### 2.5 Methods of data analysis

In this study, there three types of different statistical models were applied; the survival model to examine the determinate risk factors that affect survival time to onset of polycythemia and longitudinal model analysis was used to determinate risk factors that affect the longitudinal change of forced vital capacity separately. The joint model consists of longitudinal submodel for the change of forced vital capacity and survival submodel for the time to onset of polycythemia of chronic obstructive pulmonary patients. The data were checked, cleaned, coded, entered, and analyzed by using SPSS version 20 and R version 4.3.0 software. Bivariate logistic regression was performed to identify the potential candidate variable and each variable with a *p*‐value less than 0.05 were candidate into a multiple logistic regression analysis to determine the factors significantly associated with the Hepatitis B virus. Finally, variables with *p*‐values less than 0.05 in the multivariable logistic regression model were taken as statistically significantly.^9^

### 2.6 Exploratory data analysis

Explanatory data analysis can serve to determine as much of the information about raw data as likely, plotting each graphs to prudently determine the data should be achieved first before any formal model fitting is carried out. Hence, this investigation is explored the data by using descriptive statistics and profile plot of FVC over the period of the study and asses the nature of the data by discovering individuals profile and the average progression. The single profile graphs and the variance structure are used to improvement understanding of the variability in the data and to control which random effects to be measured in the linear the linear mixed model. The mean structure was used to gain intuition on the time function that can be used to model the data.^10^ Furthermore, the Kaplan−Meier estimator was used to estimation and graph survival probabilities as a function of time and the observed difference between survival probabilities of predictor variables were tested using log‐rang test.^11^

### 2.7 Linear mixed model for longitudinal data

Multiple observations of the same subject across time give rise to the longitudinal data analysis. When measurements are done on the similar subject at several times and when quantities are taken on related topics, longitudinal response data may arise. In both cases, the results (responses) variables are likely to be associated that is, the measuring forced vital capacity repeatedly through the duration of the study, would introduce linear mixed effect model for the analysis of continuous longitudinal responses. Whereas the longitudinal modeling between specific subject variations on forced vital capacity was completed to understand difference among individuals, the continuous model inside subjects differences were employed to analyze change over time.^12^

LMM is extending from classical linear regression model that takes in to account both fixed effect random effect terms. The random effect contains subject specific random effect and the fixed effect contains the set of predictors that are fixed across the subject or the same for all subjects. The fixed effect factors in the Linear mixed model reflect the population‐wide associations between the predictions and the forced vital capacity. Because random effects are specific to individuals within a population, they are directly employed to simulate the unpredictable variation in the forced vital capacity across various data levels.

### 2.8 Survival data analysis

The response variable of interest in survival analysis is the amount of time until an event happens. The term “survival analysis” refers to methods where the data being examined is the amount of time it takes for a particular event of interest to occur. As opposed to the use of other statistical techniques, it is most significant when there exist censoring data. It entails the modeling and analysis of data, with the time until polycythemia occurs among COPD as the primary end point (time‐to‐event data). Time is defined as the number of months or observational time in month between the start of a person's follow‐up and the occurrence of an event.^13^

### 2.9 Joint model analysis for longitudinal and survival data

The join consists of two linked submodels, the measurement model for the longitudinal process (forced vital capacity), and the to event (time to onset of polycythemia) model for the survival model for the survival process. The joint modeling approach used to obtain the less bias and more efficient inference.^14^

## 3 RESULT AND DISCUSSION

### 3.1 Descriptive analysis

The baseline sociodemographic and clinical traits of the patent of the patient participants enrolled in the analysis are shown in Table 2 below.

**Table 2:** Summary statistics for independent variables.

Variables	Categories	*N* (%)	Censored	Event
Sex	Male	148 (55.6%)	88	60
	Female	118 (44.4%)	96	22
Residence	Rural	120 (45.1%)	89	31
	Urban	146 (54.9%)	95	51
Marital‐status	Single	62 (23.3%)	40	22
	Married	115 (43.3%)	84	31
	Widowed	52 (19.5%)	38	14
	Divorce	37 (13.9%)	22	15
Smoking	Nonsmoker	188 (70.7%)	156	32
	Smoker	78 (29.3%)	28	50
Occupation	Farmer	76 (28.6%)	55	21
	Merchant	82 (30.8%)	56	26
	Government employed	40 (15.0%)	22	18
	Others	68 (25.6%)	51	17
Education	No educated	108 (40.7%)	88	20
	Primary educated	86 (32.3%)	58	28
	Secondary educated	36 (13.5%)	21	15
	Diploma and above	36 (13.5%)	17	19
Comorbidities	No	150 (56.4)	119	31
	Yes	116 (43.6)	65	51
HIV	No	233 (87.6%)	168	65
	Yes	33 (12.4%)	16	17

	Description of continuous variables			
	Minimum	Maximum	Mean	**Standard deviation**
Age at baseline	18	86	47.03	14.71
Weight at baseline	41	74	57.28	7.44
FVC	53	94	74.45	8.59

Abbreviation: FVC, forced vital capacity.

From the result among 266 COPD patients treated at the University of Gondar from February 1, 2019 to February 1, 2022, 184 (69.17%) were censored, whereas the remaining 82 (30.83%) patients were events.

The table below shows that the population was composed of 266 patients with COPD drug treatment follow‐up, of which 148 (55.6%) were males and 146 (54.9%) lived in urban areas. About 116 (43.6%) had comorbidity disease and 33 (12.4%) of COPD patients had HIV. About 78 (29.3%) are COPD patients having smoking status and regarding marital status 62 (23.3%), 115 (43.3%), 52 (19.5%), and 37 (13.9%) were single, married, widowed, and divorced, respectively. Regarding educational status 108 (340.7%), 86 (32.3%), 36 (13.5%), and 36 (13.5%) patients were not educated, primary educated, and secondary educated, respectively. About 76 (28.6%), 82 (30.8%), 40 (15.0%), and 68 (25.6%) patients were farmers, merchants, government workers, and other fields occupations, respectively.

The average forced vital capacity among chronic obstructive pulmonary patients was 74.45 with a standard deviation of 8.59. The mean age of chronic obstructive pulmonary patients at baseline was 47.03 years with a standard deviation of 14.71. The mean weight of chronic obstructive pulmonary patients at baseline was 57.28 kg with a standard deviation of 7.44.

Based on the result of Table 3, all longitudinal effects, the random intercept, and the slope model was the one with the smallest AIC and BIC values. The intercept and slope can vary randomly between individuals with random intercept and random slope models. This demonstrates that each patient's forced vital capacity for COPD was unique at the baseline and fluctuated randomly from a visit to visit.

**Table 3:** Selection of random effects.

Random effect model for FVC			
	AIC	BIC	Loglik
Random intercept	9936.26	9957.8	−4964.13
Random slope	9923.65	9950.57	−4956.83
Random intercept and slope	9898.39	9942.61	−4930.2

Abbreviations: AIC, Akaki information criteron; BIC, Bayesian information criterion; FVC, forced vital capacity.

Table 4 displays that the null model was the model fitted without covariates whereas the full model was the model fitted with all covariates considered for the model. Therefore, the full model was a better fit for the data due to the small AIC and BIC.

**Table 4:** Linear mixed model comparisons.

Model selection for FVC			
	AIC	BIC	Loglik
Full model	9936.26	9957.8	−4964.13
Null model	10,128.56	10,220.08	−5047.278

Abbreviations: AIC, Akaki information criteron; BIC, Bayesian information criterion; FVC, forced vital capacity.

Based on Table 5 univariable analysis the variable follow‐up visiting time of the patient, smoking habit, the palace of residence, marital status, HIV, education, weight, related disease (comorbidities), and sex were predictors variables which are significant at 25% level in the univariable analysis can be candidates for multivariable statistical analysis.

**Table 5:** **Univariable analysis for linear mixed effects model**.

Effect	Categories	Estimate	SE	*t* Value	*p* Value
Smoking (nonsmoker)	Smoker	4.32162	0.9603134	4.50022	<0.0001
Age		−0.02495	0.0309398	−0.80645	0.4207
Education (ref = no educated)	Primary education	4.39658	0.9296773	4.72914	0.0000
	Secondary education	2.75974	2.0177397	1.36774	0.1726
	Diploma and above	0.72679	1.8856788	0.38542	0.7002
Residence (ref = rural)	Urban	2.84740	0.8961314	3.17744	0.0017
Marital status (ref = single)	Married	−2.48387	1.1035580	−2.25078	0.0252
	Widowed	−2.67611	1.4391655	−1.85949	0.0641
	Divorce	−3.82206	1.9341499	−1.97610	0.0492
Sex (ref = female)	Male	2.01180	0.9069654	2.21817	0.0274
Comorbidities (ref = no)	Yes	2.87373	0.8978988	3.20050	0.0015
HIV (ref = no)	Yes	4.42157	1.5517345	2.84943	0.0047
Occupation (ref = farmer)	Merchant	0.14752	1.1750029	0.12555	0.9002
	Government worker	−1.36193	1.4485952	−0.94017	0.3480
	Other	−0.15645	1.2345006	−0.12673	0.8993
Weight		0.00340	0.068140	0.049863	0.9603
Visit time		−0.15216	0.0395091	−3.85116	<0.0001

Results from the above Table 6 showed that the smoking status of the patient, presence of related diseases (comorbidities), HIV, education status of patients, and weight was positive statistically significant predictors for longitudinal change of forced vital capacity. However, time visit was negatively associated for the longitudinal change of forced vital capacity among COPD outpatients at. On the other hand, marital status, age, occupation, sex, and residence of COPD patients were not significant. Moreover, from the random effect estimates table, the estimated subject‐specific variability was statistically significantly at 95% confidence level. The statistical significance of this parameter supports the assumption of heterogeneous variances for the repeated measurement data.

**Table 6:** Parameter estimates for the final linear mixed effects model.

Estimation for fixed effect						
Variables	Categories	Estimate	SE	95%	CI	*p* Value
				Lower	Upper	
Intercept		62.282	3.884	54.678	69.843	0.0000
Education (ref = no educated)	Primary education	2.843	0.996	0.883	4.777	0.0047
	Secondary education	1.887	2.003	−2.025	5.826	0.3470
	Diploma and above education	−0.666	1.843	−4.271	2.954	0.7182
Marital status (ref = single)	Married	−2.011	1.189	−4.338	0.319	0.0920
	Widowed	−1.051	1.376	−3.755	1.635	0.4455
	Divorce	−1.311	1.912	−5.060	2.434	0.4933
Residence (ref = rural)	Urban	0.821	0.943	−0.950	2.722	0.3849
Sex (ref = female)	Male	0.96	0.931	−0.858	2.784	0.3033
Comorbidities (ref = no)	Yes	2.183	0.876	0.467	3.901	0.0133
HIV (ref = no)	Yes	4.825	1.476	1.936	7.724	0.0012
Smoking (ref = nonsmoker)	Smoker	2.790	1.035	0.750	4.802	0.0075
Weight		0.178	0.068	0.045	0.311	0.0091
Visit time		−0.153	0.038	−0.227	−0.077	0.0001

Estimates for random effects						
**Cov parm**	**Stdanard deviation (estimate)**	**95% CI**				
		**Lower**	**Upper**			
Intercept (*b*_0*i*_)	7.3888534	6.677	8.167			
Visit time (*b*_1*i*_)	0.498	0.436	0.568			
Residual	3.9930	3.828	4.165			
Corr (*b*_0*i*_, *b*_1*i*_)	−0.54					

Abbreviation: ref, reference category of categorical variables.

### 3.2 Kaplan−Meier survival curves

**Figure 1:**
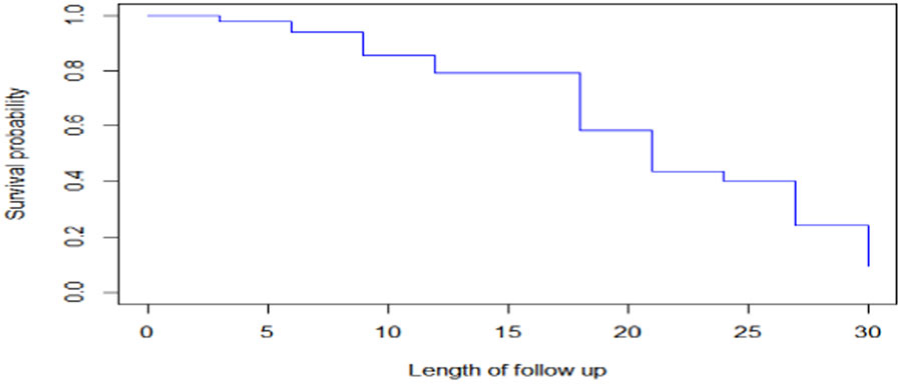
The overall estimate of Kaplan−Meier survival function plot of chronic obstructive pulmonary disease patients.

Figure 1 showed that the Kaplan−Meier survival curves for each study variable provide an initial insight into the shape of the survival function. The parts of the plot revealed the overall survival probability of COPD patients and it was a nonincreasing step function with the corresponding increment in the survival time.

#### 3.2.1 Exploring the survival time of COPD patients with categorical variables

**Figure 2:**
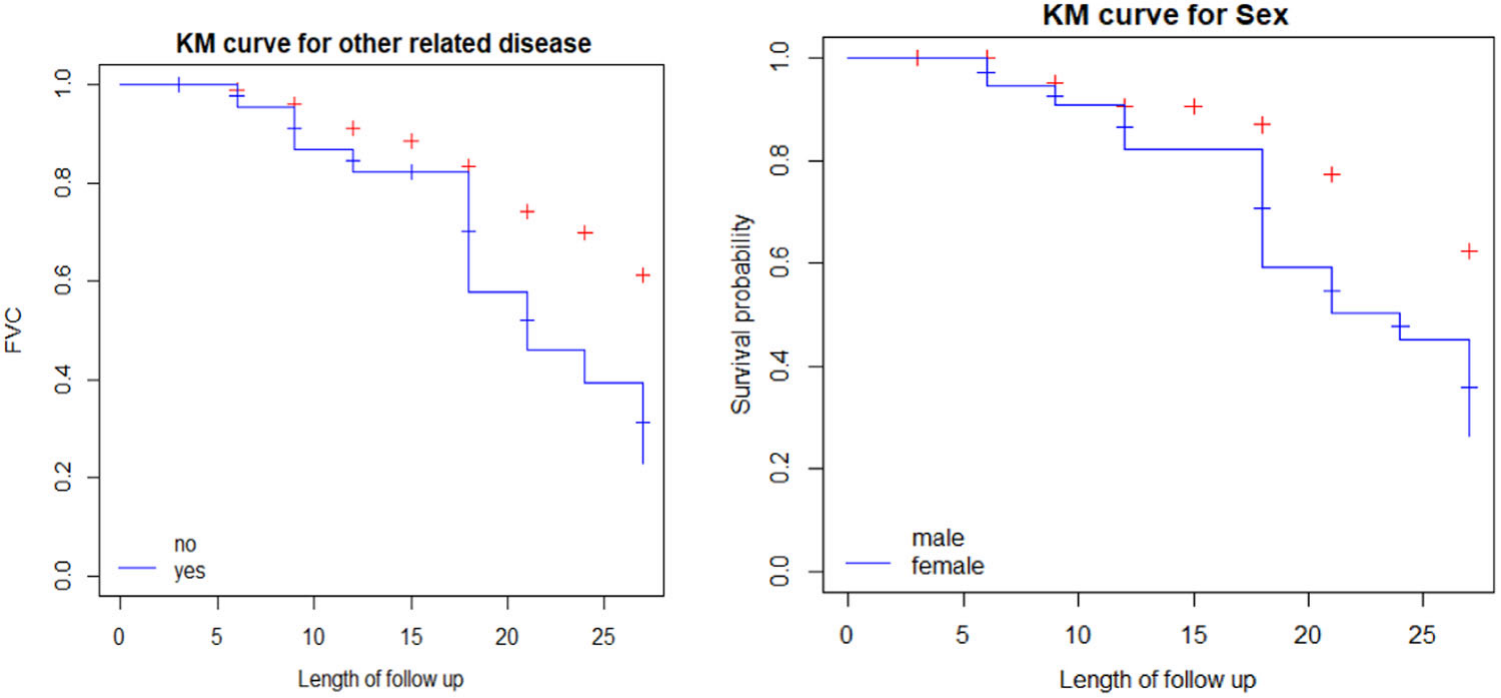
Time to onset of polycythemia by categorical variables of COPD patients. COPD, chronic obstructive pulmonary disease; KM, Kaplan−Meier.

Figure 2 displayed the time to onset of polycythemia of COPD patients by categorical variables in the study. The Kaplan−Meier survival curve indicates whether there is a difference in time to default between different categories of the variables. (1) The plot of the Kaplan−Meier curve for comorbidities of COPD patients indicates that the survival probability of patients who had no comorbidities is high as compared to those who had comorbidities patients. (2) Plot of Kaplan−Meier curve for males shows that the survival probability of males is higher as compared to female COPD patients.

The above Table 7 provided that comorbidities, sex, education, smoking, marital status, and HIV COPD patients had a statistically significant difference in the survival experience of these patients (to onset polycythemia) between their different categories at 5% of the level of significance. On the other hand, no statistically significant difference was observed in the survival experience of patients based on their residence and occupation.

**Table 7:** Log‐rank test for each categorical variable in the study.

Variable	*χ* ^2^	*df*	*p* Value
Sex	12.4	1	<0.0001
Comorbidities	13.8	1	<0.0001
Education	17.3	3	<0.0001
Residence	3	1	0.08
Occupation	3.9	3	0.3
HIV	22.3	1	<0.0001
Smoking	38.5	1	<0.0001
Marital status	13.5	3	0.004

### 3.3 Model selection and Cox PH assumption

#### 3.3.1 Cox PH assumption

The PH assumption asserts that the hazards ratios are constant over time.

That means the risk of failure must be the same no matter how long the subjects have been followed. To test this assumption the GLOBAL test and schonfield residuals were performed.

From above Table 8, it was clear to see that the *p*‐value of GLOBAL test was statistically insignificant. This indicated that the PH assumption was not violated.

**Table 8:** Proportional hazards assumption.

Variable	*χ* ^2^	*df*	*p* Value
Sex	0.738	1	0.390
Residence	2.719	1	0.099
Occupation	2.934	3	0.402
Comorbidities	1.288	1	0.256
Weight	0.584	1	0.445
HIV	0.197	1	0.657
Age	2.674	1	0.102
Smoking	2.629	1	0.105
Marital status	2.469	3	0.481
Education	2.163	3	0.539
GLOBAL	22.422	16	0.130

Abbreviation: df, degree freedom.

From the above Table 9, age of patient, place of residence, comorbidities, HIV status, education, occupation, smoking status, sex of patients, weight of patients, and marital status were statistically significant at 25% level of significance and taken as candidate variables for multivariable Cox PH model. On the other hand, no one was statistically insignificant predictors at 25% level of significance for the univariable Cox PH model.

**Table 9:** **Univariable analysis for Cox proportional hazard model**.

Variables	Categories	Estimate	SE	*Z* Value	*p* Value
Sex (ref = female)	Male	0.8102	0.2399	3.377	0.0007
Comorbidities (ref = no)	Yes	0.7798	0.2176	3.584	0.0003
Residence (ref = rural)	Urban	0.4164	0.2213	1.881	0.0599
Occupation (farmer)	Merchant	0.5218	0.2872	1.817	0.0692
	Government worker	0.5447	0.3366	1.619	0.1055
	Other	0.4394	0.3108	1.414	0.1575
Age		−0.024354	0.007356	−3.311	0.0009
Smoking (ref = nonsmoker)	Smoker	1.2927	0.2155	5.999	<0.0001
Marital status (ref = single)	Married	0.1611	0.2518	0.640	0.5222
	Widowed	−0.9707	0.4524	−2.146	0.0319
	Divorce	−0.9391	0.4091	−2.296	0.0217
Weight		0.03346	0.01746	1.916	0.0554
HIV (ref = no)	Yes	1.384	0.281	4.923	<0.0001
Education	as. factor (education)	0.9768	0.2533	3.856	0.0001

Abbreviations: ref, reference category for predictor variable; SE, standard error.

Results from Table 10 revealed sex of patient, comorbidities (related diseases), HIV, smoking, occupation, weight, and marital status were positively statistical significant associated predictors for time to onset of polycythemia. However, age of patients was negatively associated with time to onset of polycythemia. On the other hand, residence of patient and educational status were not statistically significant predictors for the time to onset of polycythemia among COPD patients at 5% level of significance.

**Table 10:** Result of the final Cox PH model for the time to onset of polycythemia of COPD patients.

Variable	Categories	Estimate	SE	HR	CI for 95% of HR	*p* Value
Sex (ref = female)	Male	1.20680	0.33644	2.651	(1.340−5.247)	0.0003
Residence (ref = rural)	Urban	−0.06953	0.27325	0.848	(0.487−1.478)	0.799
Occupation (ref = farmer)	Merchant	1.26343	0.33959	2.237	(1.138−4.340)	0.0002
	Government worker	0.56324	0.40028	2.199	(0.045−3.625)	0.159
	Other	1.48438	0.38281	2.598	(1.208−5.586)	0.0001
Comorbidities (ref = no)	Yes	0.91095	0.26073	2.185	(1.295−3.686)	0.0005
Weight		0.06309	0.01939	1.070	(1.029−1.114)	0.0011
HIV (ref = no)	Yes	1.55493	0.35029	3.599	(1.775−7.296)	<0.0001
Age		−0.02669	0.01140	0.986	(1.008−1.965)	0.0192
Smoking (ref = nonsmoker)	Smoker	1.34164	0.27931	4.457	(2.549−7.794)	<0.0001
Marital status (ref = single)	Married	1.08350	0.36827	2.503	(1.175−5.333)	0.0033
	Widowed	0.81013	0.56893	1.778	(0.558−5.661)	0.1544
	Divorce	1.59067	0.67934	3.173	(1.763,13.20)	0.0192
Education (no = educated)	Primary education	0.62191	0.33577	1.680	(0.837−3.372)	0.064
	Secondary education	0.75265	0.61560	1.607	(0.412−6.261)	0.2215
	Diploma and above education	0.28485	0.46775	1.557	(0.625−3.878)	0.5425

Abbreviations: COPD, chronic obstructive pulmonary disease; ref, reference category for categorical predictors; SE, standard error.

#### 3.3.2 Joint modeling analysis for longitudinal and survival data

Results from Table 11 showed that comorbidities, HIV, smoking, and educational status were positively associated with the longitudinal change of forced vital capacity. However, marital status, weight, and visit time were negatively associated with the longitudinal change of forced vital capacity in the longitudinal submodel. The result also shows that smoking, comorbidities, and HIV were positively associated with the time to onset of polycythemia. However, marital status, sex of patients, occupation, and weight were negatively associated with the time to onset of polycythemia among COPD patients in the survival submodel. Therefore smoking, comorbidities, marital status, weight, and HIV jointly affected the two responses, which are change of FVC and time to onset of polycythemia among COPD patients. There is evidence of a relationship between the forced vital capacity and the onset of polycythemia in patients with COPD, based on an estimate of the association parameter (*α*) is negative (−0.006) indicating that forced vital capacity is negatively associated with time to onset of polycythemia of patients from COPD treatments. This means that a decreasing trend in the forced vital capacity would be increasing the risk of polycythemia patients among COPD treatments.

**Table 11:** Parameter estimates of joint modeling.

	Longitudinal submodel			Survival submodel	
Variables	Categories	$\overset{`}{\beta }(\text{SE}(\overset{`}{\beta }))$	*p* Value	$\overset{`}{\beta }(\text{SE}(\overset{`}{\beta }))$	*p* Value
Intercept		67.5389 (3.801)	<0.0001	4.8524 (0.5522)	<0.0001
Education (no education)	Primary education	3.0008 (0.577)	0.0029	−0.1465 (0.0963)	0.1041
	Secondary education	2.0736 (0.620)	0.2836	−0.1650 (0.1745)	0.1289
	Diploma and above education	0.0231 (1.7242)	0.9490	−0.0556 (0.1311)	0.6715
Age				0.0060 (0.0033)	0.0669
Marital status (ref = single)	Married	−2.8462 (1.3585)	0.0362	−0.2560 (0.1073)	0.0171
	Widowed	−2.9767 (1.6174)	0.0657	−0.1469 (0.1618)	0.3638
	Divorce	−3.4436 (2.1475)	0.1088	−0.0556(0.1311)	0.6715
Residence (ref = rural)	Urban	1.1937 (0.9358)	0.2021	0.0491 (0.0752)	0.5139
Sex (ref = female)	Male	0.7326 (0.9445)	0.4380	−0.3166 (0.0971)	0.0011
Comorbidities (ref = no)	Yes	2.5186 (0.8753)	0.0040	0.2103 (0.0785)	0.0074
HIV (ref = no)	Yes	5.1488 (1.5187)	0.0007	0.3494 (0.0985)	0.0004
Smoking (ref = nonsmoker)	Smoker	2.5119 (1.0518)	0.0169	0.2809 (0.0780)	0.0003
Visit time		−0.150 (0.0394)	0.0001		
Occupation (ref = farmer)	Merchant			−0.3198 (0.0962)	0.0009
	Government worker			−0.1998 (0.1100)	0.0694
	Other			−0.3643 (0.1064)	0.0006
Weight		−0.0067 (0.0704)	0.038	−0.0154 (0.0052)	0.0031
Association				0.006 (0.0048)	0. 0367
	Random effect estimate				
Effect	SE (estimate)	Corr (intr)			
Intercept	7.2523	−0.5202			
Visit time	0.4191				
Residual	3.0613				

Abbreviations: ref, reference category for predictor variable; SE, standard error.

### 3.4 Interpretation of the results

Table 11 displayed the results for the joint modeling of forced vital capacity measure and time to onset of polycythemia among COPD patients. According to the joint model result, the following interpretations presented for the parameters of the longitudinal submodel and survival submodel.

In the linear mixed effect baseline weight of the patient, education status, marital status, comorbidities, HIV, smoking status, and visit time were significantly associated with the longitudinal change forced vital capacity among COPD outpatients in the longitudinal submodel. The estimated coefficient of the fixed effect of intercept was 67.54, indicating that the average longitudinal change of forced vital capacity among COPD outpatients was 67.54% by excluding all the variables in the model. One more visit of change forced vital capacity among COPD outpatients resulted in a 0.15% decrement in the average forced vital capacity, keeping all other variables are constants. For a one‐unit increase in weight, the average forced vital capacity among COPD outpatients was significantly increased by 6.18%, keeping all other variables constant. The estimate of forced vital capacity who had married marital status was significantly lower by 2.85% compared to the single marital status of forced vital capacity among COPD patients by holding all other variables constant. The average forced vital capacity of smokers was significantly higher by 2.5119% as compared to the average forced vital capacity of nonsmokers among COPD patients keeping all other variables constant. In addition, the average forced vital capacity with HIV infected was significantly higher by 5.1488% as compared to the average forced vital capacity free from HIV infected among COPD patients keeping all other variables constant. The average forced vital capacity who had related diseases (comorbidities) was significantly higher by 2.5186 compared to the average forced vital capacity for that who had no related diseases among COPD patients, keeping all other variables constant. In addition, the average forced vital capacity that had primary educated was significantly higher by 3.001% compared to the average forced vital capacity that had no educated among COPD patients.

In the survival, the estimated hazard ratio (HR) of polycythemia for patient's weight was exp (−0.0154) = 0.98, implying that for a unit a unit increment in the weight of patients, the hazard of polycythemia among COPD patients was significantly decreased by 2% keeping all other variables constant. The estimated HR of polycythemia for patients of married marital status was exp (0.2560) = 0.11, implying that a married marital status of polycythemia is 89% times less likely to develop polycythemia as compared to single marital status among COPD outpatients keeping all variables constant. The estimated HR of polycythemia patients of sex was exp (−0.3166) = 0.73, implying that male patients of polycythemia are 27% times less likely reduction to as compared to a female of polycythemia among COPD outpatients keeping all variables constant.

The estimated HR of polycythemia for patients who were merchant occupation was exp (−0.3198) = 0.73, implying that for a merchant occupation of polycythemia is 27 times less likely reduction to occur as compared to farmers of polycythemia among COPD outpatients keeping all variables constant. In addition, the estimated HR of polycythemia for patients who were tasked in another field of work was exp (−0.03643) = 0.69, implying that for another field of occupation polycythemia is 31% times less likely reduction to occur as compared to farmers occupation of polycythemia among COPD outpatients keeping all variables constant. The estimated HR of polycythemia patients who had related disease was exp (0.2103) = 1.2334, implying that the presence of related disease of polycythemia is 23.34% times more likely to develop polycythemia as compared to the absence of related disease among COPD outpatients keeping all variables constant. In addition, the estimated HR of polycythemia patients with HIV infection was exp (0.3494) = 1.4182, implying that the presence of HIV‐infected polycythemia is 41.82 times more likely to develop polycythemia as compared to the absence of HIV among COPD outpatients keeping all variables constant. The estimated HR of polycythemia patients who patients who had a smoking habit was exp (0.2809) = 1.3243, implying that patients who had a habit of smoking polycythemia are 32.43% times more likely to onset polycythemia as compared to no smoker among COPD outpatients keeping all variables constant.

The estimated association parameter was *α* = −0.006, indicating that there is a negative association between the forced vital capacity and time to onset of polycythemia among COPD outpatients. The result indicated that the lower value for a forced vital capacity measure was associated with a higher risk of polycythemia and vice versa.

## 4 DISCUSSION

In this study, the association parameters was statistically significant in the joint model, this was an indicator of the correlation between the two responses and showed that the joint model was a better fitto the data than the separate models. This finding was consistent with another study done by Long and Mills, a study conducted by a joint model trained in a single had very good performance in discriminating among diagnosed and prediagnosed participants in the remaining test studies, which concluded that joint modeling is an improvement over traditional survival modeling because it considering all the longitudinal observation od covariates that are predictive of an event.^15^

HIV is a very important clinical predictor variable for a longitudinal measure of forced vital capacity and time to onset of polycythemia among COPD patients. The estimated HR of patients with HIV infection of polycythemia was exp (0.3494) = 1.4182, implying that patients with HIV infection of polycythemia are 41.82% times more likely to develop as compared to patients without HIV infection of polycythemia among COPD outpatients keeping all variables constant. This study confirms the study conducted by Pefura‐Yone et al.

Among patients with HIV infection in this setting and who for many have a history of pulmonary tuberculosis, the presence of the chronic respiratory symptoms and other determinants identified in this study should trigger specific investigation for a possible underlying COPD. Such a proactive approach were help optimizing the care of those patients.^6^

From the final selected model; sex, weight, smoking status, marital status, presence of related disease, patients with HIV, and occupation of patients were found to be statistically significant effects for the survival submodel. To interpret the result the HR was calculated in the survival submodels. The study by Zhang et al. revealed that the event of males was 3.60 more likely as compared to females In our study also patient's sex was found to be statistically significant with time to polycythemia and this indicates that the HR of males was (1−0.73 = 0.27) 27% times less likely as compared to females. The result was contradicted with the study done by Zhang et al. Additionally, this reveals that females were suffered to onset of polycythemia due to traditional cooking food in this study. The patient's weight was found to be a statistically significant effect with time to polycythemia and this indicates that there was a 2% increase in the expected hazard to a one unit increase in weight by adjusting the other covariates constant. The HR of smoking status was (HR = 0.76. When it is compared the hazard of smokers with none smokers, the hazard of smokers was high as compared to none smoker COPD patients. This study coincides with a study by.^16^

In the longitudinal submodel, the predictor variable like marital status, baseline age, education, comorbidities, HIV, and smoking was found to be a statistically significant effect with forced vital capacity. According to the result, the average marital status of forced vital capacity for COPD patients whose marital status was married is 89% times less than developed polycythemia as compared to single marital status. When the change of forced vital capacity level increased by one unit, the weight of patients increased by 0.0618%. This result is also in line with the findings of Wang et al. Advanced age (>60 years old) was identified as the most important risk factors for COPD (OR = 3.3). For people aged 40−59, smoking was the most the important risk factor for COPD (OR = 2.7). Among people aged 40−59, those aged 54 or, with a BMI a with a BMI of less than 23 kg/m^2^, and a smoking history of more than 33 pack‐years smoking history of more than 33 pack‐years had the highest prevalence of COPD (37.5%).^17^ The average forced vital capacity of COPD patients who smoke a cigarette was 2.5119 times more likely as compared to patients who have no habit of smoking cigarettes. This study was supported by^16^ that is, the patients who have the habit of smoking are 2.55 times more likely to have the risk as compared to patients who have no smoking habit.^16^ Almost all patients (98.8%) were smokers as compared to the non‐smokers among COPD patients. When the age of patients increased by 1 year the change of forced vital capacity of COPD patients significantly increased by 0.0928%.^16^ This study lined with a study by Lee et al. The study found that the age increased unit change the COPD patients increased by an estimated 65%.^18^

The average forced vital capacity of COPD patients who had related diseases (comorbidities) was significantly higher by 2.5186 compared to the average forced vital capacity of COPD patients who had no related diseases. The most frequently associated morbidities were arterial hypertension (59.5%), dyslipidemia (54.3%), and type 2 diabetes mellitus (31.2%); 32% of the patients suffered heart disease. There is a high prevalence of active smoking, type 2 diabetes mellitus, and heart disease in patients referred for COPD to Canary Island pneumology outpatient services. This finding lined with the other study by.^19^

## 5 CONCLUSION

In this study, the results of both separate and joint analyses were displayed. But, the use of the use of a joint model analysis compared to a separate model analysis adjusted for the correlation between the two responses presented a significant reduction in the standard errors and then provides more efficient inferences. When the overall performance of the separate and joint models was compared in terms of model parsimony and goodness of fit, the joint model performed better based on its significant likelihood ratio test. This means the joint modeling can benefit the analyses of both repeated measures of forced vital capacity with time to onset of polycythemia among chronic obstructive pulmonary outpatients.

The longitudinal submodel under the joint modeling analysis showed that age, education, comorbidities, marital status, HIV, smoking, observation time, and weight were significantly associated with the change of forced vital capacity. And also the survival submodel under the joint modeling analysis showed that marital status, sex, smoking, comorbidities, weight, HIV, and occupation were statistically significant factors for the time to onset of polycythemia among COPD patients. Additionally, the association parameter (the effect of the real unobserved longitudinal change of forced vital capacity) was also statistically significant for the onset of polycythemia in COPD patients.

Results from the joint model analysis revealed that marital status, comorbidities, smoking, HIV, and weight were significantly associated with the two responses (a repeated measure of forced vital capacity and time to onset of polycythemia) of COPD patients.

The joint model performed best overall, with less variability, the more statistical significance of the association parameters, and greater goodness of fit than the standalone model. The combined model was found to be preferable for the simultaneous analysis of longitudinal measurement and survival data, according to the authors.

## AUTHOR CONTRIBUTIONS

**Yoseph Kassa**: Conceptualization; data curation; formal analysis; investigation; methodology; validation; visualization; writing—review and editing. **Dessie Melese**: Formal analysis; methodology; supervision; validation; visualization; writing—original draft; writing—review and editing. **Anteneh Asmare**: Formal analysis; methodology; supervision; validation; visualization; writing—review and editing. **Gashu Workneh**: Formal analysis; methodology; supervision; validation; visualization; writing—review and editing.

## CONFLICT OF INTEREST STATEMENT

The authors declare no conflict of interest.

## ETHICS STATEMENT

Ethical clearance was obtained from the University of Gondar, College of Natural and Computational Science Ethical Clearance Review Committee (reference number: 02/03/974/06/2022) granted permission to use the patient's data for this study. For confidentiality, there had been no linkages with individual sufferers and all information had no private identifier and had been kept confidential.

## TRANSPARENCY STATEMENT

The lead author Dessie Melese affirms that this manuscript is an honest, accurate, and transparent account of the study being reported; that no important aspects of the study have been omitted; and that any discrepancies from the study as planned (and, if relevant, registered) have been explained.

## Data Availability

The data used to support the findings of this study are available from the corresponding author upon request.
